# Microparticle Shedding from Neural Progenitor Cells and Vascular Compartment Cells Is Increased in Ischemic Stroke

**DOI:** 10.1371/journal.pone.0148176

**Published:** 2016-01-27

**Authors:** Gemma Chiva-Blanch, Rosa Suades, Javier Crespo, Esther Peña, Teresa Padró, Elena Jiménez-Xarrié, Joan Martí-Fàbregas, Lina Badimon

**Affiliations:** 1 Cardiovascular Research Center (CSIC-ICCC), Barcelona, Spain; 2 Biomedical Research Institute Sant Pau (IIB-Sant Pau), Barcelona, Spain; 3 Department of Neurology, Hospital de la Santa Creu i Sant Pau, Barcelona, Spain; University of Technology Sydney, AUSTRALIA

## Abstract

**Purpose:**

Ischemic stroke has shown to induce platelet and endothelial microparticle shedding, but whether stroke induces microparticle shedding from additional blood and vascular compartment cells is unclear. Neural precursor cells have been shown to replace dying neurons at sites of brain injury; however, if neural precursor cell activation is associated to microparticle shedding, and whether this activation is maintained at long term and associates to stroke type and severity remains unknown. We analyzed neural precursor cells and blood and vascular compartment cells microparticle shedding after an acute ischemic stroke.

**Methods:**

Forty-four patients were included in the study within the first 48h after the onset of stroke. The cerebral lesion size was evaluated at 3–7 days of the stroke. Circulating microparticles from neural precursor cells and blood and vascular compartment cells (platelets, endothelial cells, erythrocytes, leukocytes, lymphocytes, monocytes and smooth muscle cells) were analyzed by flow cytometry at the onset of stroke and at 7 and 90 days. Forty-four age-matched high cardiovascular risk subjects without documented vascular disease were used as controls.

**Results:**

Compared to high cardiovascular risk controls, patients showed higher number of neural precursor cell- and all blood and vascular compartment cell-derived microparticles at the onset of stroke, and after 7 and 90 days. At 90 days, neural precursor cell-derived microparticles decreased and smooth muscle cell-derived microparticles increased compared to levels at the onset of stroke, but only in those patients with the highest stroke-induced cerebral lesions.

**Conclusions:**

Stroke increases blood and vascular compartment cell and neural precursor cell microparticle shedding, an effect that is chronically maintained up to 90 days after the ischemic event. These results show that stroke induces a generalized blood and vascular cell activation and the initiation of neuronal cell repair process after stroke. Larger cerebral lesions associate with deeper vessel injury affecting vascular smooth muscle cells.

## Introduction

Circulating microparticles (cMPs) are phospholipid blebs sized 0.1–1.0μm shed from the plasma membrane of eukaryotic cells when injured, activated, or undergoing apoptosis. cMPs are shed from several cell types, and have been shown to reflect cellular activation and/or tissue degeneration occurring in vivo [1]. High endothelial and platelet MP levels have been observed in patients who have had an acute ischemic stroke [2], severe hypertension and increased risk of coronary heart disease [3–5]. Due to their molecular cargo cMPs seem to contribute to both vascular disease initiation and progression and also be involved in its clinical outcomes. cMPs may have relevant clinical applications including their potential use both as biomarkers of disease for improving cardiovascular risk prediction [6] and as novel therapeutic targets [7].

Neural precursor cells (NPCs) express CD34, a progenitor cell biomarker, and CD56 (neural cell adhesion molecule -NCAM-), a neural surface marker present in both neural stem and mature cells. NPCs have been shown to increase after stroke and migrate to the lesion site where they differentiate to mature neural cells in the rodent [8] and the human brain [9]. Thus, it seems plausible that NPCs also release MPs after brain injury. Nevertheless, NPC-derived cMPs after a stroke have never been analyzed.

Stroke is the second leading cause of death worldwide, responsible for the 9 percent of the total 50.5 million deaths each year, and the principal cause of disability in the elderly. In acute ischemic stroke the mechanisms of brain ischemia are critically dependent on endothelial, vascular and inflammatory factors [10,11]. In fact, levels of endothelial-derived MPs directly correlate with clinical disease severity and infarct volume and are markers of vascular pathology [12,13]. Nevertheless, if stroke is involved in increased MP shedding of other cells of the blood and vascular compartment (BVCCs) and NPCs, and if this activation is maintained at the long term and correlates to stroke severity still remains unclear. Therefore, we aimed to determine MP shedding from different cells at the onset of stroke and at 7 and 90 days and compare it to subjects at high risk but without cardiovascular disease. We also aimed to analyze the association between cMPs and cerebral infarction size, etiology and level of chronic disability in patients with ischemic stroke.

## Materials and Methods

### Patients

Forty-four patients with a suspected ischemic stroke were included in the study. Patients were admitted to the Neurology Department at Hospital de la Santa Creu i Sant Pau (Barcelona, Spain) and included within the first 48h after the onset of stroke. The Ethics Committee at Hospital de la Santa Creu i Sant Pau approved the study, and all patients or their legal representatives gave written informed consent. The study was conducted according to the Declaration of Helsinki.

After inclusion in the study, a medical record was administered to obtain demographic factors, medical and therapeutic data, and stroke etiological subtype, according to the SSS-TOAST classification and NIHSS score at admission. All patients underwent magnetic resonance imaging (MRI, n = 30) or non-contrast computed tomography (CT, n = 14). MRI and CT were performed on patients at a mean of 3–7 days after the presentation of stroke as part of the standard clinical care protocol. Lesion volumes were evaluated by an observer blinded to cMP quantification and calculated as (AxBxC)/2, where A is the maximum lesion diameter, B is the perpendicular of A, and C is the coronal diameter. Old lesions were not included as lesion volume. Patients were categorized in tertiles of lesion volume (1^st^ tertile: 0 to 0.08cm^3^, n = 14; 2^nd^ tertile: from 0.081 to 4.40cm^3^; n = 15 and 3^rd^ tertile: from 4.41 to 88cm^3^, n = 15). Blood samples were taken within the first 48h after the onset of stroke, and then after 7 and 90 days.

### Subjects without cardiovascular disease

The 44 control subjects included in this study were at high cardiovascular risk but free of cardiovascular disease or cancer and belong to the SAFEHEART cohort [14], an open, multicentre, long-term prospective study. Demographic and clinical characteristics data, cardiovascular history, classic cardiovascular risk factors and current treatment for hypercholesterolemia were obtained from all subjects using a standardized report form at inclusion. The study was approved by the local ethics committee and was conducted according to the Declaration of Helsinki. A written informed consent was obtained from all participants prior to the study.

### Blood sampling

Venous blood was withdrawn after 10–14 hours of fasting into 3.8% sodium citrate tubes. Blood cells were removed by low-speed centrifugation (250xg, 15min) at room temperature (RT) in order to avoid in vitro platelet activation. Platelet-rich plasma (PRP) was carefully aspirated, leaving about 1mm undisturbed layer on top of the cells. A second centrifugation step (11000×g, 10min, RT) and a third centrifugation step (11000xg, 3min, RT) were performed to ensure the complete removal of platelets and to obtain the platelet-free plasma (PFP). All samples were processed identically and within 30min after extraction. PFP aliquots of 250μL were immediately frozen in liquid nitrogen and stored at -80°C until processing for isolation and quantification of cMPs.

### Circulating microparticles isolation and quantification

To isolate cMPs, 225μL of frozen PFP aliquots were thawed on melting ice and centrifuged at 20000×g for 30min. The supernatants (200μL) were discarded and the cMP-enriched pellet was washed once with 200μL citrate-phosphate buffered saline solution (citrate-PBS; 1.4mM phosphate, 154mM NaCl, 10.9mM trisodium citrate, pH 7.4). A second equal centrifugation step was made and 200μL of the supernatant were discarded to resuspend the remaining cMP pellets in 75μL citrate-PBS.

Triple-label flow cytometric analysis was performed as described by Suades *et al* [15]. Briefly, 5μL of washed cMP suspensions were diluted in 30μL PBS buffer containing 2.5mM CaCl_2_ (Annexin Binding Buffer, ABB, BD Biosciences, San Jose). Thereafter, combinations of 5μL of CF405M-conjugated AV (Immunostep, Salamanca, Spain) with two specific monoclonal antibodies (mAb, 5μL each, S1 Table) labeled with fluorescein isothiocyanate (FITC) and phycoerythrin (PE), or the isotype-matched control antibodies were added. Samples were incubated 20min at RT in the dark and diluted with ABB before being immediately analyzed on a FACSCantoIITM flow cytometer (except for MPs from smooth muscle cells, SMC). SMC-derived cMPs were quantified according to the methodology described by Leroyer *et al* [16]. Briefly, 5μL of the MP suspension were incubated 20min at RT in the dark with 5μL AV-CF405M and 5μL CD142-FITC (tissue factor, TF) in a final volume of 50μL ABB. cMPs were fixed with 450μL ABB/PFA 2% during 30min and centrifuged at 20 000×g for 30min to pellet cMPs. After removing the supernatant, cMPs were permeabilized with 25μL of ABB/saponin 0.1% 20min at RT in the dark. After permeabilizing, 5μL of smooth muscle actin (SMA)-α-PE were added to the cMP suspension and incubated 20min at RT in the dark and finally diluted in ABB prior to flow cytometer analyses.

**Fig 1:**
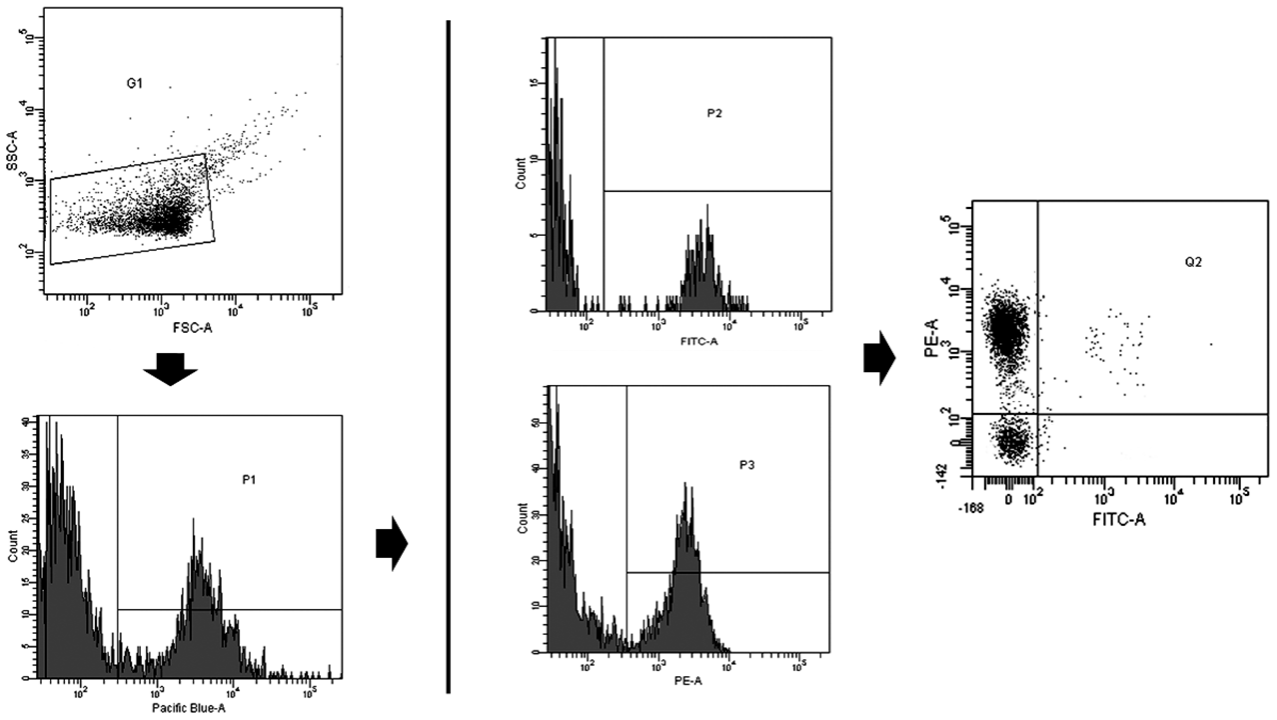
Gating and acquisition strategy for the detection of circulating microparticles by flow cytometry. Gate limits were established before analyses using the Megamix-Plus FSC beads for cytometer settings in microparticle analysis (S1 Fig). G1 was set according to cMPs size and granularity (defined as <1μm). Annexin V-CF405M^+^ cMPs (P1) were selected from G1. cMPs binding FITC^+^ (P2) or PE^+^ (P3) labeled antibodies were selected from P1 and quantified. Double staining with FITC- and PE- labeled antibodies from P1 (Annexin V^+^ cMPs) was quantified from Q2 region. Pacific blue is the channel for CF405M quantification. CF405M is a blue fluorescent dye. FITC indicates fluorescein isothiocyanate; PE, phycoerythrin.

Acquisition was performed at 1min per sample at low flow. Flow rate was measured before each experiment (mean of 17 ± 0.5μL/min). Forward scatter (FSC), side scatter (SSC) and fluorescence data were obtained with the settings in the logarithmic scale. Gate limits were established following the criteria previously described [15,17]. The upper threshold for FSC to 1μm was set with the Flow Check YG Size Range Calibration Kit (Polysciences, Warrington, PA, USA) with beads of 1μm in diameter, and with the Megamix-Plus FSC beads (BioCytex, Marseille, France). Megamix-Plus FSC beads for cytometer settings in microparticle analysis are a mix of beads of the following bead-equivalent diameters: 0.1μm, 0.3μm, 0.5μm and 0.9μm (S1 Fig). According to beads signal, the lower detection limit was placed as a threshold above the electronic background noise of the flow cytometer for FSC and at the second logarithm for SSC. cMPs within the established gate limits (>0.1 to 1μm) were identified and quantified based on their binding to Annexin V and reactivity to cell-specific mAb (Fig 1).

To identify positive marked events, thresholds of fluorescence were also set based on samples incubated with the same final concentration of isotype-matched control antibodies after titration experiments. AV binding level was corrected for autofluorescence using fluorescence signals obtained with microparticles in a calcium-free buffer (PBS). To reduce background noise, buffers were prepared on the same day and filtered through 0.2μm pore size filters under vacuum.

We quantified AV^+^ cMPs from NPC (CD56/CD34), platelets (CD61), endothelial cells (CD146), erythrocytes (CD235ab), leukocytes (CD45), lymphocytes (CD3), monocytes (CD14) and SMC (SMA-α) carrying markers of cell activation as shown in S1 Table. Other leukocyte-derived cMPs were inferred by subtracting agranulocytes cMPs (lymphocytes plus monocytes) from total leukocyte-derived cMPs instead of labeling with specific mAbs.

Data were analyzed with the FACSDivaTM software (version 6.1.3, Becton Dickinson). cMP concentration (number of cMPs per μL of PFP) was determined according to Nieuwland’s formula [18], based on sample’s volume, flow cytometer’s flow rate and the number of fluorescence-positive events (N), as follows: cMPs/μL = N x (Vf/Va) x (Vt/FR) x (1/Vi) [where Vf(μL) = final volume of washed cMP suspension, Va(μL) = volume of washed cMP suspension used for each labelling analysis, Vt(μL) = total volume of cMP suspension before fluorescence-activated cell sorting analysis, FR(μL/min) = flow rate of the cytometer at low mode (the average volume of microparticle suspension analyzed in one minute), 1 is the μL unit of volume, and Vi(μL) = original volume of plasma used for microparticle isolation].

### Statistical analysis

Sample size was determined assuming a loss of 0% participants (ENE 3.0, GlaxoSmithKline, Brentford, United Kingdom). To detect mean differences in the number of CD56^+^/CD34^+^/AV^+^ cMP of 10 units with a conservative SD of 15, 26 subjects would be needed to complete the study (α risk = 0.05, power = 0.9). However, to obtain greater statistical power, the sample size was nearly doubled. The number of CD56^+^/CD34^+^/AV^+^ cMP was used to determine the sample size but all cMPs were considered primary outcomes.

Statistical analyses were performed using the SPSS Statistical Analysis System (version 22.0). Descriptive statistics [mean ± sd, mean ± sem, or n (%)] were used to describe the baseline characteristics of the patients and the outcome variables. Variables with a skewed distribution were transformed to their natural logarithms for analyses.

To analyze the changes in cMPs after 7 and 90 days of the onset of stroke, repeated measures ANOVA with the Bonferroni post-hoc test was used. One-way ANOVA and the Bonferroni post-hoc test were used to compare the differences of the outcome variables in response to the etiology of stroke and other pathological conditions. Repeated measures ANCOVA and the Bonferroni post-hoc test were used to compare the differences of changes in outcome variables in response to the etiology of stroke and other pathological conditions. ROC-Curve analyses for predicted probabilities were performed to identify the threshold concentration of cMPs able to discriminate between patients and controls, and the corresponding area under the curve (AUC) with its 95% confidence interval (CI) was calculated. A cut-off level of cMPs was determined with the shortest distance from upper left corner of the ROC curve, minimizing [(1-sensitivity)^2^ + (1-specificity)^2^]. Correlation analyses were performed with the Spearman’s rho correlation coefficient. P was considered significant when <0.05.

## Results

### Baseline characteristics

Baseline characteristics of control patients at high cardiovascular risk and stroke patients can be found on S1 Appendix and S2 Fig.

According to the SSS-TOAST classification, 6 patients had suffered a large artery atherosclerosis stroke; 13 suffered a cardioembolic stroke; 7 patients had a small vessel occlusion stroke; 2 suffered a stroke of uncommon etiology and 16 subjects suffered a stroke of undetermined etiology.

### Levels and changes of neural precursor cell-derived circulating microparticles

**Fig 2:**
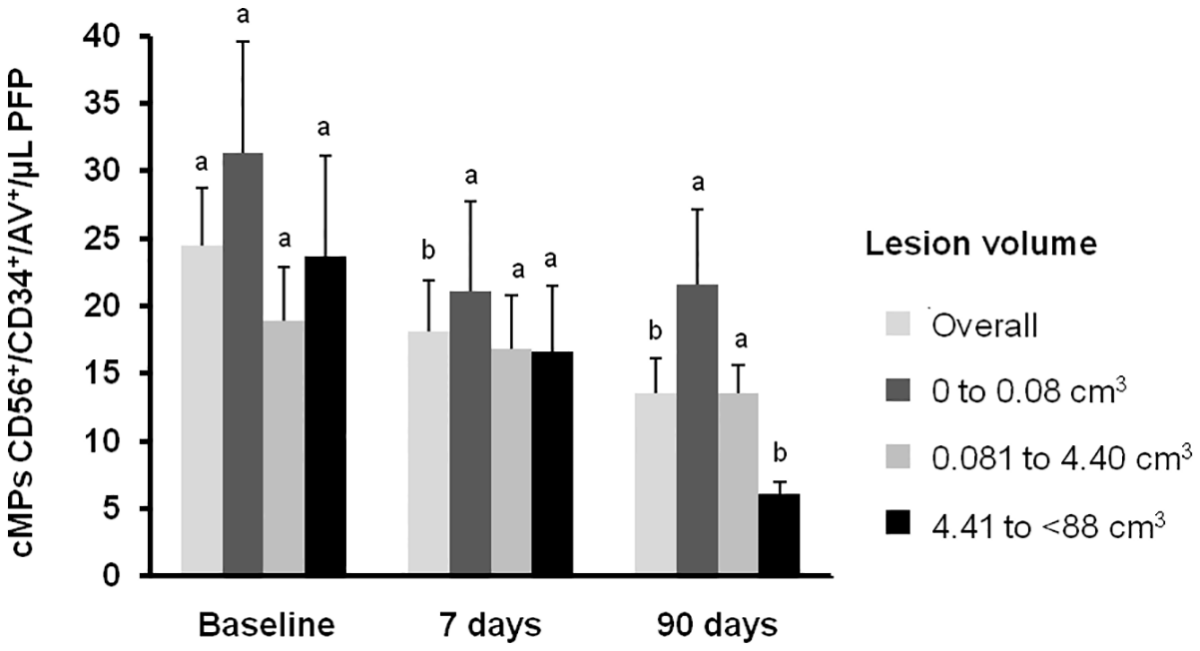
Circulating microparticles CD56^+^/CD34^+^/AV^+^ of patients at the onset of stroke and at 7 and 90 days by tertiles of lesion volume. Results are represented as mean ± sem. Different letters within tertiles of lesion volume denote statistical differences, measured by repeated measures ANCOVA with the lesion volume as the covariate and the Bonferroni post-hoc test. cMPs denotes circulating microparticles; PFP, platelet free plasma and AV, Annexin V.

Compared to high risk controls, patients had increased number of NPC-derived cMPs at the stroke onset, and after 7 and 90 days (P<0.001). A ROC-curve analysis (S3 Fig) showed that CD56^+^/CD34^+^/AV^+^ cMPs at a cut-off point of 2.8 cMPs/μl of PFP, *P*<0.0001, properly discriminated between controls and stroke patients with a 81.8% sensitivity and 83.3% specificity [area under de curve (AUC) = 0.894 (95% CI 0.823, 0.965)]. As depicted in Fig 2, NPC-MPs were found decreased compared to baseline levels at 7 (P = 0.050) and 90 days (P = 0.008) of the onset of stroke in overall patients. In addition, patients in the upper tertile of lesion volumes presented lower NPC-originated cMP concentration at 90 days than patients in the lower tertile (P = 0.007 for the comparison between the 1st and 3rd tertile, one-way ANOVA with the Bonferroni post-hoc test). Moreover, NPC-derived cMPs at 90 days after the onset of stroke negatively correlated to lesion volumes (P = 0.001, -0.478, Spearman correlation coefficient). Patients with any level of disability (modified Rankin Scale ≥1 at 90 days after the onset of stroke) presented 2.9 fold lower levels of NPC-derived cMPs (P = 0.001) than patients without any level of disability. No significant correlation was found between NPC-derived cMPs and the stroke etiology according to the SSS-TOAST classification, and between NPC-derived cMPs and the NIHSS score at any time.

### Levels of circulating microparticles from the cells of the blood and vascular compartment

As shown in Table 1, compared to high cardiovascular risk subjects, patients showed higher levels of cMPs originated from platelets, endothelial cells, erythrocytes and leukocytes, including monocytes, lymphocytes and other leukocytes. Moreover, these differences still remained after 7 and 90 days of the onset of stroke (data not shown). No significant differences were found between patients and controls in cMPs derived from SMC at the onset of stroke and after 7 days. At 90 days after the onset of stroke, SMA-α^+^/AV^+^ cMPs were increased in stroke patients compared to controls (P = 0.046, one-way ANOVA).

**Table 1:** cMP levels in the 44 non-CVD controls and the 44 patients at the onset of stroke.

cMPs (cMP/μL PFP)	CONTROLS (n = 44)	PATIENTS (n = 44)	*P*
*MP from all cell origins*			
AV^+^	343.10 ± 173.70	693.56 ± 324.83	<0.001
*MP from neural progenitor cells*			
CD34^+^/CD56^+^/AV^+^	1.70 ± 2.46	24.48 ± 25.73	<0.001
*MP from platelets*			
CD61^+^/AV^+^	104.69 ± 66.90	176.72 ± 113.03	<0.001
CD61^+^/CD142^+^/AV^+^	5.57 ± 11.52	22.96 ± 28.13	<0.001
CD62P^+^/AV^+^	10.12 ± 8.79	39.61 ± 36.00	<0.001
*MP from endothelial cells*			
CD146^+^/AV^+^	0.01 ± 0.01	2.82 ± 4.30	<0.001
CD62E^+^/AV^+^	39.42 ± 31.05	102.70 ± 81.15	<0.001
CD146^+^/CD62E^+^/AV^+^	0.06 ± 0.39	2.21 ± 3.93	0.002
*MP from erythrocytes*			
CD235ab^+^/AV^+^	75.27 ± 59.75	149.37 ± 84.51	<0.001
*MP from leukocytes*			
CD45^+^/AV^+^	44.39 ± 30.71	120.57 ± 68.63	<0.001
CD62L^+^/AV^+^	31.43 ± 17.83	87.80 ± 79.16	<0.001
CD11-α^+^/AV^+^	48.22 ± 49.61	120.67 ± 112.53	<0.001
*MP from lymphocytes*			
CD3^+^/AV^+^	2.58 ± 4.26	8.75 ± 10.73	<0.001
*MP from other leukocytes*			
CD45^+^/ CD3^-^/CD14^-^/AV^+^	35.76 ± 28.34	80.48 ± 57.78	<0.001
*MP from monocytes*			
CD14^+^/AV^+^	4.86 ± 4.69	20.05 ± 24.13	0.001
CD11-α^+^/CD14^+^/AV^+^	0.32 ± 1.29	3.48 ± 5.74	0.003
CD142^+^/CD14^+^/AV^+^	0.82 ± 2.20	4.61 ± 7.69	0.005
*MP from smooth muscle cells*			
SMA-α ^+^/AV^+^	21.81 ± 27.78	23.70 ± 25.12	0.835
CD142^+^/SMA-α ^+^/AV^+^	1.10 ± 1.92	7.79 ± 10.17	<0.001
*MP from activated cells*			
CD29^+^/AV^+^	47.12 ± 40.34	87.88 ± 61.74	<0.001
CD15^+^/AV^+^	18.06 ± 18.13	60.44 ± 84.61	<0.001
CD29^+^/CD15^+^/AV^+^	1.65 ± 2.80	16.13 ± 26.89	0.001
CD63^+^/AV^+^	2.94 ± 3.27	11.05 ± 14.79	<0.001
CD11b^+^/AV^+^	17.26 ± 15.78	39.02 ± 36.38	0.001
CD63^+^/CD11b^+^/AV^+^	0.23 ± 0.71	3.54 ± 8.22	0.005
CD142^+^/AV^+^	54.65 ± 56.23	115.01 ± 107.00	<0.001

Results are expressed as mean ± sd. Used controls were patients at high cardiovascular disease who have never suffered a stroke. Selected markers were CD56/CD34 for neural progenitor cells, CD61 for platelets, CD146 for endothelial cells, CD235ab for erythrocytes, CD45 for total leukocytes, and CD3 for lymphocyte, CD14 for monocyte origins accounting for agranulocytes and SMA-α for smooth muscle cells. Other leukocytes were inferred subtracting agranulocytes subpopulation from leukocytes fraction. The other CDs were used as biomarkers of cell activation (see S1 Table). *P* value from the one-way ANOVA.

In addition, patients also presented higher levels of cMPs carrying markers of cell activation (CD142^+^, CD62L^+^, CD62E^+^, CD62P^+^, CD11-α^+^, CD29^+^, CD15^+^, CD63^+^ and CD11b^+^) than non-CVD. Again, these differences still persisted after 7 and 90 days of the onset of stroke (data not shown).

### Association between circulating microparticles and stroke etiology

**Fig 3:**
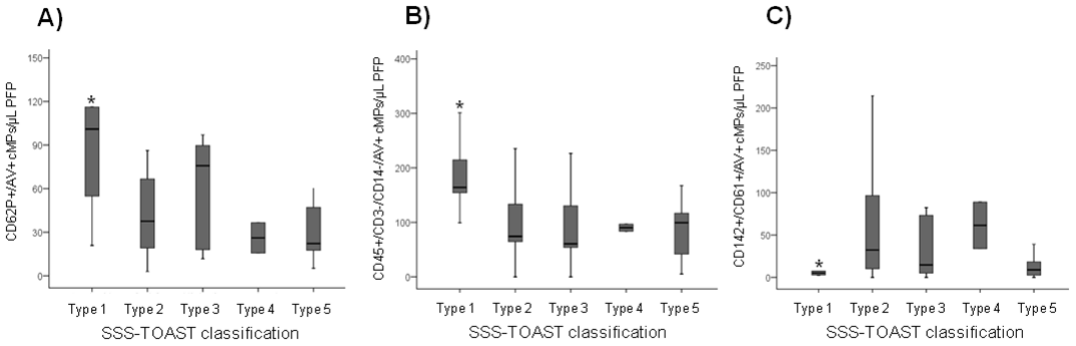
Circulating microparticles at 90 days after the onset of stroke according to the SSS-TOAST classification. A, CD62P^+^ (P-Selectin) cMPs; B, Other leukocyte-derived cMPs (CD45^+^/CD3^-^/CD14^-^) and C, Platelet-derived tissue factor positive (CD142^+^/CD61^+^) cMPs. *Significantly different from the other types of stroke (*P* from the one-way ANOVA with the Bonferroni posthoc test). cMPs denotes circulating microparticles; PFP, platelet free plasma and AV, Annexin V. Type I (n = 6) large artery atherosclerosis stroke; type II (n = 13) cardioembolic stroke; type III (n = 7) small vessel occlusion stroke; type IV (n = 2) stroke of uncommon etiology and type V (n = 16) stroke of undetermined etiology (SSS-TOAST classification).

We observed that the etiological subtype of stroke, according to the SSS-TOAST classification, was associated to differential MP shedding at 90 days (Fig 3), but not at the onset of stroke or after 7 days. Compared with the other etiologies, patients with large-artery atherosclerosis stroke (n = 6), presented higher P-Selectin (CD62P^+^/AV^+^)- carrying and other leukocyte-derived (CD45^+^/CD14^-^/CD3^-^/AV^+^) cMPs (P = 0.004 and 0.002 respectively), and lower platelet-derived cMPs carrying TF (CD61^+^/CD142^+^/AV^+^, P = 0.033) at day 90 after the stroke (one-way ANOVA). Nevertheless, the differences in cMPs between the onset of stroke and after 90 days in these patients (with large-artery atherosclerosis stroke) only reached statistical significance for CD62P^+^/AV^+^ and other leukocyte-derived cMPs (P = 0.049 and 0.047, respectively, repeated-measures ANCOVA with the Bonferroni posthoc test).

No differences in the concentration of cMPs at the onset of stroke or after 7 and 90 days or in time-course changes of the other cMPs quantified were observed between different stroke etiologies.

### Changes in circulating microparticles after 7 and 90 days of the onset of stroke

**Fig 4:**
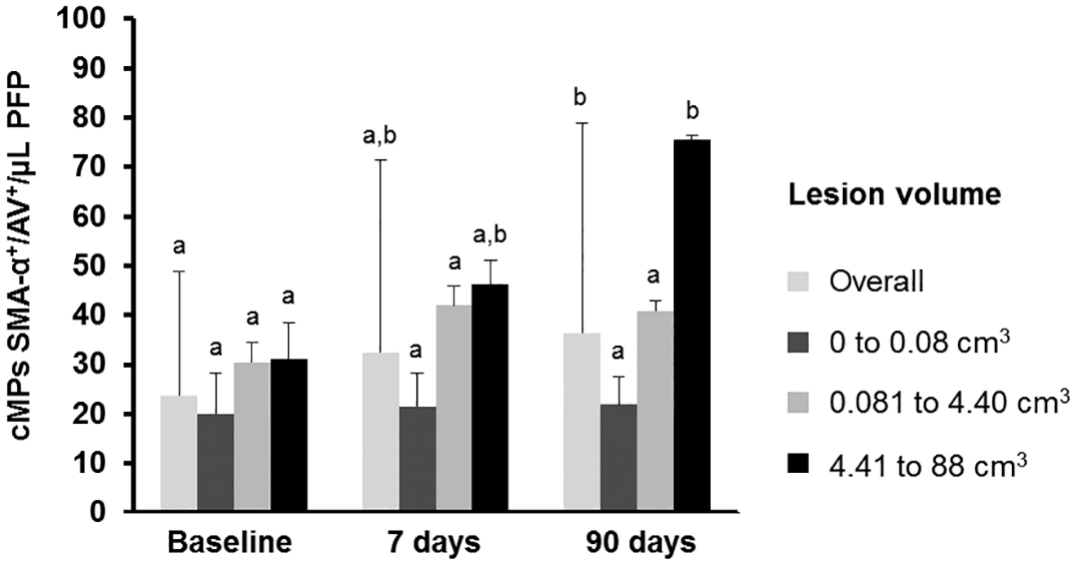
Changes in smooth muscle cell derived circulating microparticles after 90 days of stroke by tertiles of lesion size. Results are represented as mean ± sd. Different letters within tertiles of lesion volume denote statistical differences, measured by repeated measures ANCOVA with the lesion volume as the covariate and the Bonferroni post-hoc test. cMPs denotes circulating microparticles; PFP, platelet free plasma and AV, Annexin V. SMA-α (smooth muscle actin-α) was used as a biomarker of smooth muscle cells.

Changes in circulating microparticles after 7 and 90 days of the onset of stroke are shown in Table 2. CD63^+^/AV^+^ (platelet- and/or leukocyte-derived cMPs) decreased (P = 0.023) and CD63^+^/CD11b^+^/AV^+^ (leukocyte-derived cMPs) tended to decrease (P = 0.050) at 90 days. On the opposite, as depicted in Fig 4, patients in the 1st and 2nd tertile of ischemic brain size showed unaltered concentrations of SMA-α^+^/AV^+^ cMPs while patients in the highest tertile of cerebral lesion volumes showed increased SMC-derived cMPs after 90 days of the onset of stroke (P = 0.025 for the differences between 90 days and baseline, repeated-measures ANCOVA with the Bonferroni posthoc test).

**Table 2:** cMPs at the onset of stoke and after 7 and 90 days in the 44 patients included in the study.

cMPs (cMP/μL PFP)	STROKE ONSET	7 DAYS	90 DAYS	*P*
*MP from all cell origins*				
AV^+^	702.86 ± 63.00	758.12 ± 80.86	838.68 ± 100.97	0.224
*MP from platelets*				
CD61^+^/AV^+^	176.72 ± 113.03	213.34 ± 109.74	187.66 ± 139.69	0.301
CD61^+^/CD142^+^/AV^+^	22.96 ± 28.13	32.48 ± 44.64	31.75 ± 45.85	0.140
CD62P^+^/AV^+^	39.61 ± 36	51.65 ± 36.71	52.54 ± 50.14	0.087
*MP from endothelial cells*				
CD146^+^/AV^+^	2.82 ± 4.30	3.99 ± 5.99	3.62 ± 5.17	0.096
CD62E^+^/AV^+^	102.70 ± 81.15	155.35 ± 166.34	111.18 ± 83.53	0.142
CD146^+^/CD62E^+^/AV^+^	2.21 ± 3.93	2.25 ± 4.13	2.31 ± 3.6	0.980
*MP from erythrocytes*				
CD235ab^+^/AV^+^	149.37 ± 84.51	154.09 ± 92.05	143.15 ± 99.96	0.884
*MP from leukocytes*				
CD45^+^/AV^+^	120.57 ± 68.63	157.06 ± 119.07	139.54 ± 83.15	0.099
CD62L^+^/AV^+^	87.80 ± 79.16	100.29 ± 91.86	75.39 ± 48.16	0.103
CD11-α^+^/AV^+^	120.67 ± 112.53	116.82 ± 102.26	83.55 ± 55.53	0.293
*MP from lymphocytes*				
CD3^+^/AV^+^	8.75 ± 10.73	9.92 ± 12.99	7.22 ± 9.29	0.295
*MP from other leukocytes*				
CD45^+^/ CD3^-^/CD14^-^/AV^+^	80.48 ± 57.78	104.85 ± 78.19	96.68 ± 66.8	0.159
*MP from monocytes*				
CD14^+^/AV^+^	20.05 ± 24.13	22.44 ± 25.39	21.00 ± 25.89	0.234
CD11-α^+^/CD14^+^/AV^+^	3.48 ± 5.74	5.17 ± 8.73	3.28 ± 6.57	0.365
CD142^+^/CD14^+^/AV^+^	4.61 ± 7.69	4.71 ± 6.83	4.24 ± 7.76	0.304
*MP from smooth muscle cells*				
CD142^+^/SMA-α ^+^/AV^+^	7.79 ± 10.17	7.36 ± 13.13	12.85 ± 21.74	0.193
*MP from activated cells*				
CD29^+^/AV^+^	87.88 ± 61.74	115.15 ± 86.75	92.44 ± 87.15	0.253
CD15^+^/AV^+^	60.44 ± 84.61	51.46 ± 48.75	40.96 ± 42.73	0.368
CD29^+^/CD15^+^/AV^+^	16.13 ± 26.89	11.12 ± 19.32	11.32 ± 23.41	0.986
CD63^+^/AV^+^	11.05 ± 14.79^a^	16.16 ± 19.62^a^	9.34 ± 12.72^b^	**0.023**
CD11b^+^/AV^+^	39.02 ± 36.38	46.39 ± 47.08	38.80 ± 40.17	0.455
CD63^+^/CD11b^+^/AV^+^	3.54 ± 8.22	3.46 ± 6.02	1.31 ± 3.96	0.050
CD142^+^/AV^+^	115.01 ± 107	112.09 ± 109.65	105.57 ± 99.44	0.876

Results are expressed as mean ± sd. Used markers were CD61 for platelet, CD146 for endothelial cell, CD235ab for erythrocytes, CD45 for total leukocytes, and CD3 for lymphocyte, CD14 for monocyte origins accounting for agranulocytes and SMA-α for smooth muscle cells. Other leukocytes were inferred subtracting agranulocytes subpopulation from leukocytes fraction. The other CDs were used as biomarkers of cell activation (see S1 Table). *P* value from the repeated measures ANOVA with the Bonferroni post-hoc test. Values in rows with different superscript letters are significantly different.

No differences were observed in the levels of the other cMPs after 7 or 90 days after the onset of stroke. Notwithstanding, after 7 days of the onset of stroke, intravenous thrombolysis (IVT)-treated patients (n = 10) had 59% less platelet-originated cMPs (CD61^+^/AV^+^ cMPs, P = 0.014). Nevertheless, after 90 days of the onset of stroke, these differences disappeared.

## Discussion

To our knowledge, little is known about chronic consequences of stroke in cell activation. We observed that the number of NPC-derived cMPs is increased in stroke patients with respect to high cardiovascular risk controls, and declines with time post-event only in those patients with higher lesion volumes.

NPC-derived (CD56^+^/CD34^+^/AV^+^) cMPs have not been measured before. NPCs can secrete several factors that regulate neurogenesis and modulate inflammatory responses after brain damage, and they are mobilized at sites of injury to replace dead neurons [8,9] and repair the blood-brain barrier [19]. As cMPs are considered both biomarkers and cell-cell communication messengers [20], it seems plausible that NPC-derived MP shedding may reflect ongoing brain repair processes after ischemic injury since is increased at the onset of stroke when the brain tissue is damaged, and maintained in those patients with lower lesion volumes. NPC-derived cMPs decline gradually the days after the ischemic event (especially in patients with higher lesion volumes), although the levels do not achieve those of subjects without a previous cerebrovascular event, independently of the brain lesion size. The overall results suggest that NPC-originated cMPs may be a novel biomarker of stroke. These MP may be involved in neurorepairing process as their parental cells, considering the negative correlation between lesion volumes and NPC-derived cMPs and also considering the fact that patients with any level of disability (modified Rankin Scale ≥1 at 90 days after the onset of stroke) have decreased NPC-derived cMPs at 90 days after the onset of stroke compared to patients without disability and small cerebral lesions.

Except for SMC-derived MPs, we observed that MPs from all cell origins and activation markers are increased in stroke patients compared to the non-CVD population (in a mean range of 2 to 15-fold), and this is maintained at 90 days. It suggests that increased MP shedding is secondary to both cell activation and also cell apoptosis due to vascular injury, primarily in those patients with a lesion volume >4.41cm^3^. On the other hand, increased MP shedding in acute stroke has been previously observed, especially from endothelial cells [2,13,17,21,22]. In fact, we observed that activated endothelial cell-derived MPs (CD146^+^/AV^+^ and CD146^+^/CD62E^+^/AV^+^) are the most increased cMPs (by ~300 fold, compared to the mean range of 2- to 15-fold increase in the other cMPs) in stroke patients compared to high-CV risk controls. No correlation was found between E-Selectin (CD62E)-carrying cMPs and the time of stroke onset, supporting the idea that stroke promotes chronic cell activation. These observations suggest that endothelial cMPs are biomarkers of stroke, and further suggest that activated endothelial cMPs are major players in stroke induction and vascular insult rather than in vascular repair as previously suggested [23]. We found higher levels of endothelial-derived cMPs carrying CD62E (a biomarker of endothelial activation) than CD146 (one of the most specific marker of endothelial cell lineage). It has been previously described [24] that that the expression and proportion of cell surface molecules on endothelial-derived MPs may differ from their parental cells. Therefore, it can be possible that endothelial cells, when activated, release higher amounts of MP loaded with CD62E than MP carrying CD146.

Although endothelial MPs have been previously reported to correlate with lesion volume [12,13], we only found that SMC-derived cMPs at 90 days correlate with infarction size. Correlations of cMPs with ischemic lesion volumes at presentation may suggest a correlation with the degree of endothelial apoptosis and inflammation within the ischemic lesion, while correlation of ischemic lesion with cMPs at 90 days after the presentation of stroke may reflect the long-term vascular damage after the cerebrovascular event.

TF is crucial for the initiation of coagulation, and platelet-derived MPs have 50- to 100-fold higher procoagulant activity than activated platelets [25]. Therefore, elevated levels of circulating MPs and TF-loaded cMPs are associated with an increased risk of thrombosis and thromboembolic events. Recently it has been observed that in 73 patients with an acute ischemic stroke, TF-carrying cMPs were elevated compared to healthy individuals [26], in agreement with our findings. Besides, SMC shed TF-loaded MP with thrombogenic properties in human [27] and rat aorta [28]. Along this line, platelet and SMC-derived TF^+^-cMPs were significantly elevated compared to controls in the three time points, partially explaining the increased risk of recurrent stroke in those patients.

SMC in blood vessel wall play a key role in cerebral blood flow control, brain perfusion maintenance and regulation. Surprisingly, we observed that at the onset of stroke and after 7 days, SMC-derived cMPs (but not SMC-derived MPs carrying TF) were similar to the levels observed in non-CVD controls. At 90 days however, these MP were increased compared to both controls and patients at the onset of stroke, but only in those patients with the highest lesion volumes. These results indicate that larger cerebral lesions associate with deeper vessel injury affecting smooth muscle cells function. Although SMC represent the major cell type in blood vessels, very little is known about SMC-derived MPs [16,27,28]. It is known that stroke induces SMC to switch to a phenotype through different mechanisms that will be either detrimental or beneficial to brain repair [29,30], which may be also related to MP shedding, considering that the fluctuation observed in SMC-derived MP shedding was determined by the infarct size.

In view of the results, further studies are required to elucidate if MP in stroke are procoagulatory or vasoconstrictive (one of the causes), or if they are merely the consequence of tissue damage.

This study is not exempt of limitations. Although there is increasing evidence that some MP may be formed without externalization of PS [31], in our study, MP carrying cell-specific antigens but not binding AV (a PS ligand) only accounted for <1% of the total MP population. Therefore, we only analyzed MP containing AV in their surface. Blood samples were taken within the 48h of the onset of stroke, sometimes after IVT or other medication administration because informed consent had to be obtained first. Thus, correlations between clinical parameters and MP levels could reflect delayed apoptotic cell injury and inflammatory stimulation after ischemic stroke. During the first days after the onset of stroke, patients were polymedicated. This circumstance may partially explain the fact that the main differences were observed after 90 days of the onset of stroke. Although the number of patients is relatively small, this cohort has a representative distribution of clinical characteristics, and the incidence of the different types of stroke was comparable to previously reported data [32]. Finally, this is an observational study and the meaning and pathophysiological consequences of the increase of cMPs are unclear and further research is warranted.

## Conclusions

Ischemic stroke increases MP shedding and is chronically maintained, which may in part explain the increased risk of recurrent stroke in these patients, since a previous cerebral ischemic event is one of the major risk factors for a further one. To our knowledge, this is the first time that MP shedding from several cellular origins after stroke is measured. The specific mechanism underlying the involvement of elevated MPs in stroke (cause or consequence) and their relationship with repair of ischemic tissue remains to be established to better understand stroke pathophysiology. Finally, whether specific MPs can help to clinically differentiate stroke type and affected cerebral lesion size deserve further investigation in a larger number of patients.

## Supporting Information

S1 Appendix Baseline characteristics of the patients included in the study. (PDF)

S1 Fig Gate limits for microparticle analysis with the Megamix-Plus FSC beads for cytometer settings in microparticle analysis. A) Gate limits were established before analyses using the Megamix-Plus FSC beads for cytometer settings in microparticle analysis (BioCytex, Marseille, France). According to Megamix-Plus FSC beads signal, the lower limit of quantification is >0.1μm, as beads of 0.1μm were negative for FITC signal. B) Gate limits for microparticle quantification (G1) were set according to beads signal.
(PDF)

S2 Fig Cell sources of MP for controls and patients at the onset of stroke. Pie-charts showing distribution of cMPs from controls (n = 44) and patients at the onset of stroke (n = 44) by major cell origins, indicated by percentages of each marker relative to cell lineage. Used controls were patients at high cardiovascular disease who have never suffered a stroke. Selected markers were CD61 for platelets, CD146 for endothelial cells, CD45 for total leukocytes, CD3 for lymphocytes, CD14 for monocytes and SMA-α for smooth muscle cells origins. Other leukocyte cMPs were positive for CD45 but negative for CD3 or CD14.
(PDF)

S3 Fig ROC curve analysis to determine the threshold of NPC-derived cMPs that discriminates between patients and controls. CD56^+^/CD34^+^/AV^+^ cMPs at a cut-off point of 2.8 MP/μL of PFP, *P*<0.0001, properly discriminated between controls and stroke patients with a 81.8% sensitivity and 83.3% specificity [area under de curve (AUC) = 0.894 (95% CI 0.823, 0.965)]. Used controls were patients at high cardiovascular disease who have never suffered a stroke. cMPs denotes circulating microparticles; PFP, platelet free plasma and AV, Annexin V.
(PDF)

S1 Table Cell surface molecules for circulating microparticle identification and characterization. mAb indicates monoclonal antibody; PS, phosphatidylserine; FITC, fluorescein isothiocyanate; PE, phycoerythrin; LPS, lipopolysaccharide.
(PDF)

S2 Table Baseline characteristics of subjects devoid of cardiovascular disease (n = 44) and patients at the onset of stroke (n = 44). Results are expressed as mean ± sd or n (%) when indicated. Used controls were patients at high cardiovascular disease who have never suffered a stroke. *P* value from one-way ANOVA for quantitative variables and from Chi-square analysis for qualitative variables.
(PDF)
