# Supplementary material for: Microparticle Shedding from Neural Progenitor Cells and Vascular Compartment Cells Is Increased in Ischemic Stroke
Source: PLoS One. 2016 Jan 27;11(1):e0148176. doi: 10.1371/journal.pone.0148176 (PMC4729528; doi:10.1371/journal.pone.0148176)
Supplement: S1 Appendix — (PDF) [file pone.0148176.s001.pdf]

S1 Appendix

Baseline characteristics

Patients had a mean age of 70 years, and about 66% were men. A 20% were current smokers, nearly 64% were hypertensive and about 30% were diabetic. Patients with stroke had a previous history of ischemic disease as 20% presented ischaemic cardiopathy, 5% peripheral vascular disease, 9% a previous transient ischemic attack, and 16% a previous cerebral infarction (**S2 Table**). After admission at the Hospital, nearly 23% of the patients were administered intravenous thrombolytics (IVT), and no patient suffered a second cardiovascular event while hospitalized. The non-CVD subjects included in the study presented a mean age of 73 years old, and 34% of them were men. An 18% were current smokers, nearly 50% were hypertensive and about 14% were diabetic. None of them presented ischaemic cardiopathy, peripheral vascular disease, previous transient ischemic attack or previous cerebral infarction.
