# Supplementary material for: Microparticle Shedding from Neural Progenitor Cells and Vascular Compartment Cells Is Increased in Ischemic Stroke
Source: PLoS One. 2016 Jan 27;11(1):e0148176. doi: 10.1371/journal.pone.0148176 (PMC4729528; doi:10.1371/journal.pone.0148176)
Supplement: S1 Fig — A) Gate limits were established before analyses using the Megamix-Plus FSC beads for cytometer settings in microparticle analysis (BioCytex, Marseille, France). According to Megamix-Plus FSC beads signal, the lower limit of quantification is >0.1μm, as beads of 0.1μm were negative for FITC signal. B) Gate limits for microparticle quantification (G1) were set according to beads signal. (PDF) [file pone.0148176.s002.pdf]

**S1 Figure.** Gate limits for microparticle analysis with the Megamix-Plus FSC beads for cytometer settings in microparticle analysis.

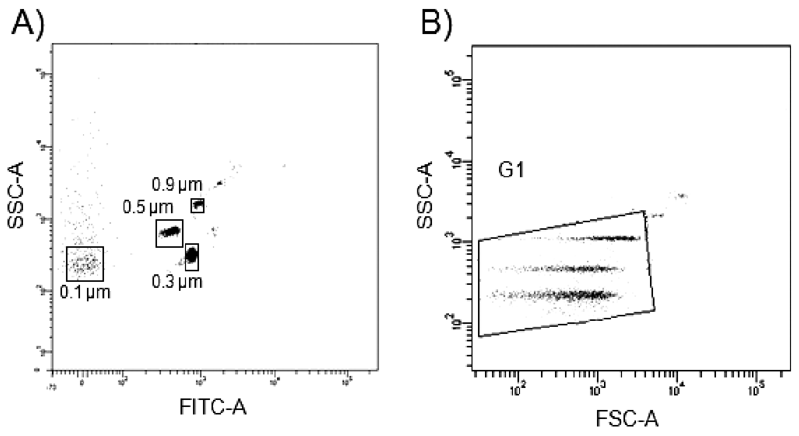


A) Gate limits were established before analyses using the Megamix-Plus FSC beads for cytometer settings in microparticle analysis (BioCytex, Marseille, France). According to Megamix-Plus FSC beads signal, the lower limit of quantification is >0.1µm, as beads of 0.1µm were negative for FITC signal. B) Gate limits for microparticle quantification (G1) were set according to beads signal.
