# Supplementary material for: Microparticle Shedding from Neural Progenitor Cells and Vascular Compartment Cells Is Increased in Ischemic Stroke
Source: PLoS One. 2016 Jan 27;11(1):e0148176. doi: 10.1371/journal.pone.0148176 (PMC4729528; doi:10.1371/journal.pone.0148176)
Supplement: S2 Fig — Pie-charts showing distribution of cMPs from controls (n = 44) and patients at the onset of stroke (n = 44) by major cell origins, indicated by percentages of each marker relative to cell lineage. Used controls were patients at high cardiovascular disease who have never suffered a stroke. Selected markers were CD61 for platelets, CD146 for endothelial cells, CD45 for total leukocytes, CD3 for lymphocytes, CD14 for monocytes and SMA-α for smooth muscle cells origins. Other leukocyte cMPs were positive for CD45 but negative for CD3 or CD14. (PDF) [file pone.0148176.s003.pdf]

S2 Figure. Cell sources of MP for controls and patients at the onset of stroke.

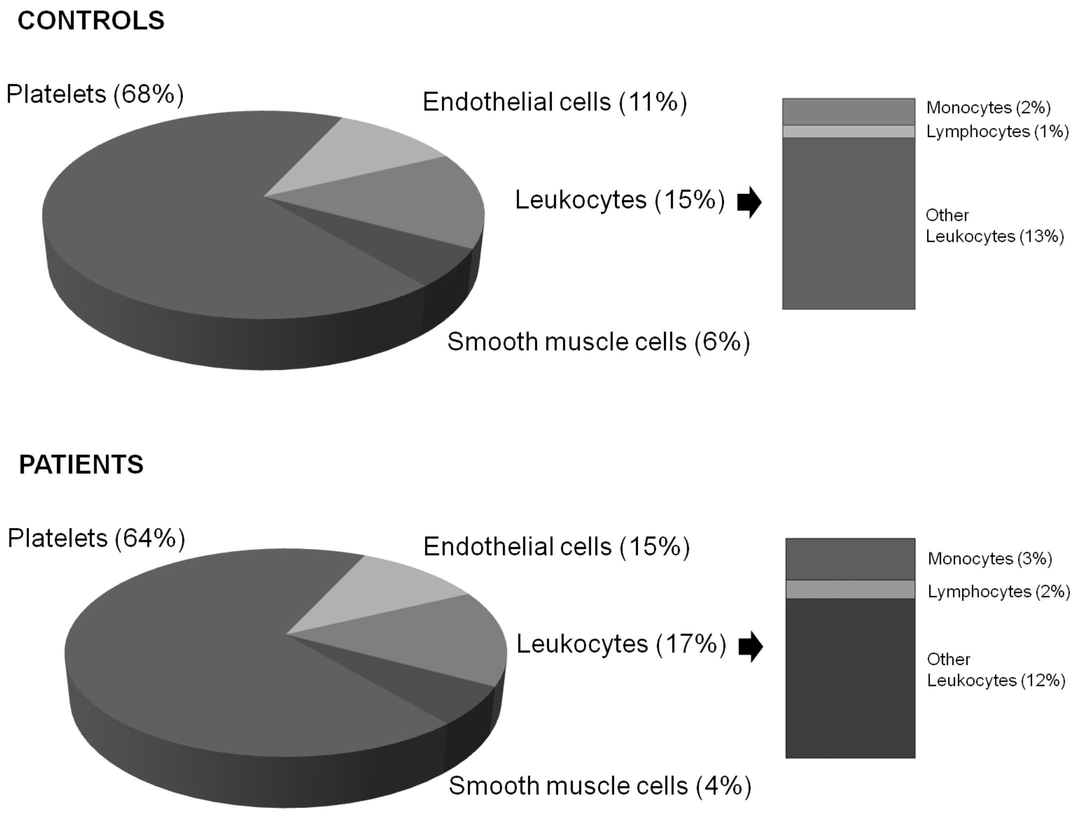

Pie-charts showing distribution of cMPs from controls (n=44) and patients at the onset of stroke (n=44) by major cell origins, indicated by percentages of each marker relative to cell lineage. Used controls were patients at high cardiovascular disease who have never suffered a stroke. Selected markers were CD61 for platelet, CD146 for endothelial cells, CD45 for total leukocytes, CD3 for lymphocyte, CD14 for monocyte and SMA-α for smooth muscle cells origins. Other leukocyte cMPs were positive for CD45 but negative for CD3 or CD14.
