# Supplementary material for: Microparticle Shedding from Neural Progenitor Cells and Vascular Compartment Cells Is Increased in Ischemic Stroke
Source: PLoS One. 2016 Jan 27;11(1):e0148176. doi: 10.1371/journal.pone.0148176 (PMC4729528; doi:10.1371/journal.pone.0148176)
Supplement: S3 Fig — CD56+/CD34+/AV+ cMPs at a cut-off point of 2.8 MP/μL of PFP, P<0.0001, properly discriminated between controls and stroke patients with a 81.8% sensitivity and 83.3% specificity [area under de curve (AUC) = 0.894 (95% CI 0.823, 0.965)]. Used controls were patients at high cardiovascular disease who have never suffered a stroke. cMPs denotes circulating microparticles; PFP, platelet free plasma and AV, Annexin V. (PDF) [file pone.0148176.s004.pdf]

**S3 Figure.** ROC curve analysis to determine the threshold of NPC-derived cMPs that discriminates between patients and controls.

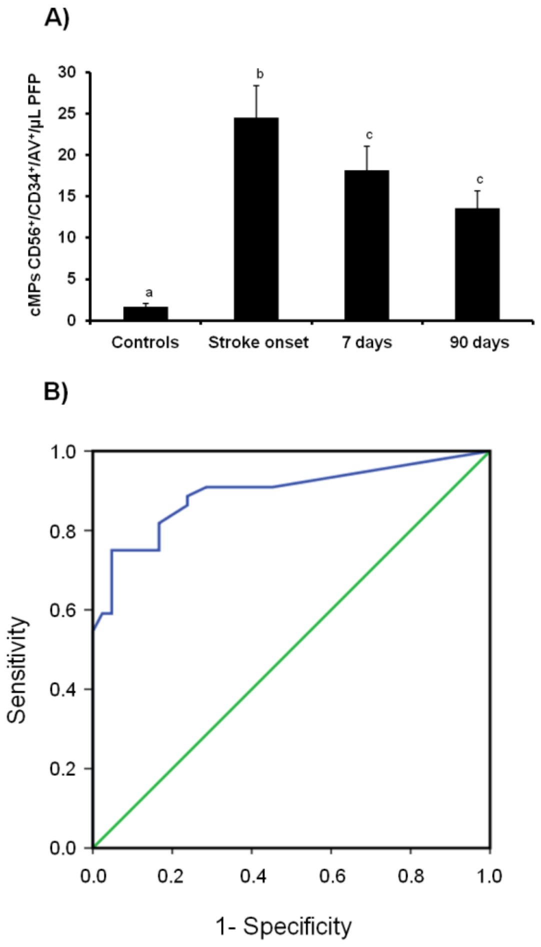


$CD56^{+}/CD34^{+}/AV^{+}$ cMPs at a cut-off point of 2.8 MP/µL of PFP, $P < 0.0001$, properly discriminated between controls and stroke patients with a 81.8% sensitivity and 83.3% specificity [area under de curve (AUC) = 0.894 (95% CI 0.823, 0.965)]. Used controls were patients at high cardiovascular disease who have never suffered a stroke. cMPs denotes circulating microparticles; PFP, platelet free plasma and AV, Annexin V.
