# Supplementary material for: Microparticle Shedding from Neural Progenitor Cells and Vascular Compartment Cells Is Increased in Ischemic Stroke
Source: PLoS One. 2016 Jan 27;11(1):e0148176. doi: 10.1371/journal.pone.0148176 (PMC4729528; doi:10.1371/journal.pone.0148176)
Supplement: S1 Table — mAb indicates monoclonal antibody; PS, phosphatidylserine; FITC, fluorescein isothiocyanate; PE, phycoerythrin; LPS, lipopolysaccharide. (PDF) [file pone.0148176.s005.pdf]

S1 Table. Cell surface molecules for circulating microparticle identification and characterization.

mAb	Aterantive name	Expression	Conjugation	Clone	Company
Annexin V	PS-binding protein	Widely expressed	CF405	--	Immunostep
IgG1γ	--	--	FITC/PE	X40	BD Biosciences
IgG1k	--	--	FITC/PE	MPOC21	BD Pharmingen
CD56	Neural Cell Adhesion Molecule	Leukocytes	FITC	B-A19	Immunotools
CD34	Mucosialin	Progenitor cells (Hematopoietic)	PE	4H11[APG]	Immunotools
CD142	Tissue Factor	Widely expressed	FITC	VD8	Sekisui diagnostics
CD61	β_3-integrin	Platelets	PE	VI-PL2	BD Pharmingen
CD62L	L-Selectin	Leukocytes	PE	LT-TD180	Immunotools
CD146	Melanoma Cell Adhesion Molecule	Endothelial Cells	FITC	P1H12	BD Pharmingen
CD62E	E-Selectin	Endothelial Cells	PE	68-5H11	BD Pharmingen
CD62P	P-Selectin	Activated Platelets	PE	AK-4	BD Pharmingen
CD235ab	Glycoporin A and B	Erythrocytes	FITC	HIR2	Immunotools
CD3	T-cell co-receptor	T-Lymphocytes	FITC	HIT3b	Immunotools
CD45	Leukocyte Common Antigen	Leukocytes	PE	MEM-28	Immunotools
CD11a	Lymphocyte Function-Associated Antigen 1	Leukocytes	FITC	HI111	Immunotools
CD29	Integrin β-1	Leukocytes	FITC	HI29a	Immunotools
CD15	Sialyl Lewis X	Leukocytes	PE	MEM-158	Immunotools
CD63	Tetraspanin 30	Activated cells	FITC	MEM-259	Immunotools
CD11b	Macrophage-1 Antigen (Mac-1)	Neutrophils, leukocytes	FITC	MEM-174	Immunotools
CD14	LPS-receptor	Macrophages, monocytes	PE	M5E2	BD Pharmingen
SMA-α	Smooth Muscle Actin α	Smooth muscle cells	PE	1A4	R&D Systems

mAb indicates monoclonal antibody; PS, phosphatidylserine; FITC, fluorescein isothiocyanate; PE, phycoerythrin; LPS, lipopolysaccharide.
