# Supplementary material for: Microparticle Shedding from Neural Progenitor Cells and Vascular Compartment Cells Is Increased in Ischemic Stroke
Source: PLoS One. 2016 Jan 27;11(1):e0148176. doi: 10.1371/journal.pone.0148176 (PMC4729528; doi:10.1371/journal.pone.0148176)
Supplement: S2 Table — Results are expressed as mean ± sd or n (%) when indicated. Used controls were patients at high cardiovascular disease who have never suffered a stroke. P value from one-way ANOVA for quantitative variables and from Chi-square analysis for qualitative variables. (PDF) [file pone.0148176.s006.pdf]

S2 Table. Baseline characteristics of subjects devoid of cardiovascular disease (n = 44) and patients at the onset of stroke (n = 44).

	CONTROLS	PATIENTS	*P*
Age (years)	73.36 ± 5.48	70.14 ± 12.29	0.595
Current smokers [n (%)]	8 (18.1)	9 (20.4)	0.748
Type 2 Diabetes Mellitus [n (%)]	6 (13.6)	13 (29.5)	0.071
Hypertension [n (%)]	22 (50)	28 (63.6)	0.199
Ischemic cardiomyopathy [n (%)]	0 (0)	9 (20.4)	0.001
Peripheral vascular disease [n (%)]	0 (0)	2 (4.5)	0.150
Previous transient ischemic attack [n (%)]	0 (0)	4 (9.1)	0.037
Previous cerebral infarction [n (%)]	0 (0)	7 (15.9)	0.006
Cerebral infarction at inclusion [n (%)]	0 (0)	44 (100)	<0.001
Stroke etiology (SSS-TOAST classification), [n (%)]			
Large artery atherothrombosis	0 (0)	6 (13.6)	<0.001
Cardioembolism	0 (0)	13 (29.5)	<0.001
Small vessel occlusion	0 (0)	7 (15.9)	<0.001
Stroke of other uncommon etiology	0 (0)	2 (4.5)	<0.001
Stroke of undetermined etiology	0 (0)	16 (36.4)	<0.001
Weight (Kg)	69.61 ± 12.12	71.23 ± 10.56	0.534
Body Mass Index (kg/m^2)	28.51 ± 4.64	26.41 ± 3.15	0.026
Body Mass Index >29.9 kg/m^2	5 (11.4)	4 (9.1)	0.754
Systolic Blood Pressure (mmHg)	148.34 ± 24.25	153.93 ± 39.3	0.429
Diastolic Blood Pressure (mmHg)	81.07 ± 9.78	86.46 ± 18.8	0.098

Results are expressed as mean ± sd or n (%) when indicated. Used controls were patients at high cardiovascular disease who have never suffered a stroke. *P* value from one-way ANOVA for quantitative variables and from Chi-square analysis for qualitative variables.
